# Bioaccessibility of Polycyclic Aromatic Hydrocarbons (PAHs) in Grilled Meat: The Effects of Meat Doneness and Fat Content

**DOI:** 10.3390/ijerph19020736

**Published:** 2022-01-10

**Authors:** Elliyana Nadia Hamidi, Parvaneh Hajeb, Jinap Selamat, Soo Yee Lee, Ahmad Faizal Abdull Razis

**Affiliations:** 1 Food Safety Research Centre, Faculty of Food Science and Technology, Universiti Putra Malaysia (UPM), Serdang 43400, Selangor, Malaysia; ell_nadia@yahoo.com; 2 Department of Environmental Science, Aarhus University, 4000 Roskilde, Denmark; parvanehhajeb@gmail.com; 3 Laboratory of Food Safety and Food Integrity, Institute of Tropical Agriculture and Food Security, Universiti Putra Malaysia (UPM), Serdang 43400, Selangor, Malaysia; sjinap@gmail.com; 4 Department of Food Science, Faculty of Food Science and Technology, Universiti Putra Malaysia (UPM), Serdang 43400, Selangor, Malaysia; 5 Natural Medicines and Products Research Laboratory, Institute of Bioscience, Universiti Putra Malaysia (UPM), Serdang 43400, Selangor, Malaysia; leesooyee@upm.edu.my

**Keywords:** bioaccessibility, polycyclic aromatic hydrocarbons (PAHs), grilled meat, meat doneness, fat content

## Abstract

Exposure to polycyclic aromatic hydrocarbons (PAHs) through diet is gaining concern due to the risk it poses to human health. This study evaluated the bioaccessibility of PAHs contained in charcoal-grilled beef and chicken in different segments of the gastrointestinal tract (GIT) with regard to the degree of doneness and fat content of the meats. The levels of 15 PAHs in the grilled meat samples and bioaccessible fractions were determined using high-performance liquid chromatography (HPLC) equipped with PAH column, and UV and fluorescence detectors. Total PAHs were found in beef (30.73 ng/g) and chicken (70.93 ng/g) before its digestion, and different PAHs’ bioaccessibility were observed in the different segments of GIT, with the highest in the stomach followed by the small intestine, despite the relatively higher bioaccessibility of individual PAHs in grilled beef as compared to those in grilled chicken. Additionally, the PAHs’ bioaccessibility increased with the increase in the degree of doneness. Positive linear correlation was observed for the PAHs’ bioaccessibility and the fat contents of grilled meat. Overall, this study highlights the influence of meat doneness (cooking time) and fat contents on the bioaccessibility and bioaccumulation of PAHs.

## 1. Introduction

Polycyclic aromatic hydrocarbons (PAHs) are organic pollutants released into the environment through incomplete combustion of organic materials, with the emission from anthropogenic events predominant [1]. Chemically, PAHs are compounds containing only carbon and hydrogen atoms, arranged in two or more fused aromatic rings by sharing a pair of carbon atoms between the rings. These compounds are mainly colorless, white, or pale-yellow solids. PAHs are deleterious to the human body because of their toxic, mutagenic and carcinogenic properties. They alter the DNA sequence and, hence, are increasing the risk of cancer [2]. In fact, the International Agency for Research on Cancer (IARC) has classified some PAHs as known, possibly or probably (Group 1, 2A or 2B) carcinogenic to humans [3]. Among these, benzo[a]pyrene is recognized as carcinogenic to humans (Group 1), while cyclopenta[cd]pyrene, dibenz[a,h]anthracene and dibenzo[a,l]pyrene are probably carcinogenic to humans (Group 2A), and benz[j]aceanthrylene, benz[a]anthracene, benzo[b]fluoranthene, benzo[j]fluoranthene, benzo[k]fluoranthene, benzo[c]phenanthrene, chrysene, dibenzo[a,h]pyrene, dibenzo[a,i]pyrene, indeno [1,2,3-cd]pyrene and 5-methylchrysene are possibly carcinogenic to human (Group 2B). The harmful effects of PAHs depend on the route of exposure, and a mixture of two or more PAHs is likely to be more carcinogenic than an individual PAH [4].

Besides smoking and occupational exposure, diet is one of the major sources of exposure to PAHs. Raw food usually does not contain high levels of PAHs. The occurrence of PAHs in food is usually due to the food’s processing, such as drying and smoking, as well as cooking of food at high temperature, such as grilling, frying and roasting [5]. Among various kinds of food, consumption of grilled, barbecued and smoked meat serves as a major means of exposing the body to PAHs Grilled, roasted and smoked foods especially are gaining popularity nowadays, both in restaurants and at home [6]. The occurrence of PAHs in meat can result from the pyrolysis of organic matter in meat, as well as from the smoke produced by the incomplete combustion of fat in meat drippings dripped over the fire during grilling using charcoal [2]. However, the levels of PAHs formed are dependent on several factors, such as method of cooking, time, type of fuel, distance of food from the heat source and fat contents [5,7,8]. In relation to the fat contents, studies have observed the increase in PAH levels with the increase in fat contents in the food, which could mainly be due to the fat pyrolysis process [9].

In the human gastrointestinal tract (GIT), not all ingested contaminants are available for absorption. Those bound to the food matrices are not digested and, hence, are not available for intestinal absorption and will be excreted through the feces. To this end, contaminant bioaccessibility, which is defined as the fraction of contaminant released from its matrix (food or soil) during digestion, has become an important tool to measure the risk of contaminants from food [10]. Bioaccessibility of food contaminants can be assessed using an in vitro digestion model that mimics the physiological/biochemical conditions in the human GIT [10]. Regarding the bioaccessibility of PAHs, most of the studies focus on soil as the matrix, assessing human uptake of PAHs in soil [11,12,13,14], while limited study has been performed for the bioaccessibility of PAHs contained in food. Few studies have reported on the bioaccessibility of PAHs in seafood, uncooked animal-based foods and cereal after an in vitro human digestion [15,16,17,18]; however, little is known about the bioaccessibility of PAHs contained in charcoal-grilled meat in the human GIT. It is worth mentioning that different food matrices can be a factor affecting the bioaccessibility of PAHs in the human GIT system, since different dietary components such as fats and fibers will influence the bioaccessibility of PAHs [12].

In view of the effects of various factors on the occurrence of PAHs in grilled meat, and the lack of information regarding the bioaccessibility of PAHs in grilled meat, this study evaluates the bioaccessibility of PAHs in grilled beef and chicken in the different segments of the human GIT in relation to the degree of doneness of meats. The relationship between fat contents of grilled meats and the bioaccessibility of PAHs is also investigated.

## 2. Materials and Methods

### 2.1. Chemicals and Materials

The standard mixture of 15 PAHs, consisting of naphthalene (Na), acenaphthene (Ac), fluorene (F), anthracene (A), phenanthrene (Phe), fluoranthene (Fl), pyrene (P), benzo(a)anthracene (BaA), chrysene (Ch), benzo(b)fluoranthene (BbF), benzo(k)fluoranthene (BkF), benzo(a)pyrene (BaP), indeno(1,2,3-cd)pyrene (IP), benzo(ghi)perylene (BgP) and dibenzo(ah)antracene (DhA) was supplied by Supelco (Bellefonte, PA, USA). All solvents used were of HPLC grade. Acetonitrile, dichloromethane, n-hexane and petroleum ether were purchased from Merck (Darmstadt, Germany). Deionized water was obtained from PURELAB^®^ Classic Water Purification System (ELGA LabWater, High Wycombe, UK). Components used to simulate juices for the in vitro human digestion model, including potassium chloride (KCl), disodium sulfate (Na_2_SO_4_), sodium bicarbonate (NaHCO_3_) and hydrogen chloride (HCl), were also supplied by Merck (Darmstadt, Germany). Potassium thiocyanate (KSCN), sodium dihydrogen phosphate (NaH_2_PO_4_), sodium chloride (NaCl), ammonium chloride (NH_4_Cl), glucuronic acid, uric acid, mucin and pancreatin were supplied by Accot (Shah Alam, Selangor, Malaysia). Albumin from bovine serum, alpha amylase, pepsin and lipase were purchased from Sigma Aldrich (St. Louis, MO, USA). Urea, glucose and bile extract were obtained from R&M Chemicals (Essex, UK). Diatomaceous earth extraction cartridges (Extrelut, 20 mL) and refill materials were purchased from International Sorbent Technology Ltd. (Hengoed, UK). Propylsulfonic acid (PRS, 500 mg) solid-phase extraction (SPE) columns and silica gel were purchased from Silica Chemical Division (Quebec City, QC, Canada).

### 2.2. Meat Sample Preparation and Marination

The raw meat, including beef fillet and chicken breast, was purchased from a local market in Seri Serdang, Selangor and stored at −20 °C. To prepare the meat for marination and grilling, frozen raw meat was thawed at 4 °C for 6 h and cut into cubes (1 cm × 1 cm). A total of 82 meat samples were prepared. For marination, marinade was prepared according to the recipe adapted from Jinap et al. [19], as shown in Table 1. All the ingredients were purchased from a local grocery store. To prepare the marinade, ingredients were mixed and blended using a kitchen blender (Blendforce BL4291 model, Tefal, Rumilly, France) for 2 min. The beef and chicken meat samples were marinated according to the Malaysian satay preparation [19]. Bamboo sticks were used to skew the marinated meats.

### 2.3. Charcoal Grilling of Marinated Meat Samples

The marinated beef and chicken meat samples were placed on a satay-type grill (fueled by charcoal) and grilled to three different degrees of doneness (rare, medium and well-done). Grilling times and surface browning/charring were used to determine the degree of doneness. These parameters were according to the report of Jinap et al. [19] and are summarized in Table S1. When it was halfway through the cooking time, the meat samples were turned and grilling continued. The grilled meat samples were homogenized in a blender and stored at −20 °C before PAH extraction or bioaccessibility testing.

### 2.4. Bioaccessibility Test of PAHs Using In Vitro Human Digestion Model

To assess the bioaccessibility of PAHs in grilled meat samples in the different segments of the human GIT, the grilled meat samples were digested in vitro using saliva, gastric, duodenal and bile juices, adopting the method of Maulvault et al. [20]. The digestive juices were prepared accordingly, and their compositions were summarized in Table S2. Where necessary, 1 M HCl was used to adjust the pH of the digestive juices. A weight of 5 g of grilled meat samples was ground and transferred into a 50 mL centrifuge tube. For in vitro saliva digestion, 5 mL of artificial saliva was added and the mixture was stirred for 5 min at 37 °C. The saliva digestion was stopped by reducing the temperature to 4 °C. Then, the suspension was centrifuged at 7000× *g* for 10 min and the supernatant was collected as a bioaccessible fraction of saliva digestion. The particulate residue remaining in the tube was further subjected to in vitro gastric digestion by the addition of 12 mL of artificial gastric juice, and incubated for 2 h at 37 °C with constant rotation. After 2 h, the temperature of the suspension was lowered to 4 °C to stop the gastric digestion. The suspension was subjected to centrifugation at 7000× *g* for 10 min. The supernatant was collected as the bioaccessible fraction of gastric digestion. The remaining residue was next subjected to intestinal digestion, where 12 mL of artificial duodenal juice was first added, followed by 6 mL of artificial bile juice (after 5 min). After incubation at 37 °C for 2 h with constant rotation, the digestion by intestinal juice was stopped by lowering the temperature to 4 °C. After centrifugation of the suspension at 7000× *g* for 10 min, the supernatant was collected as a bioaccessible fraction of intestinal digestion. The bioaccessible fractions collected from all digestive phases were stored at −20 °C until they could be further analyzed for PAH content. A schematic representation of the in vitro digestive model is shown in Figure S1. The bioaccessibility of PAHs in different segments of the GIT can be calculated as follows:

(1) $$Bio\;\left(\%\right)=\frac{\mathrm{PAH}\;in\;bioaccessible\;fraction\;\left(µg\right)}{\mathrm{PAH}\;in\;sample\;before\;digestion\;\left(µg\right)}\times 100\%\;$$

### 2.5. Extraction of PAHs and Clean-Up

Sample preparations for determination of PAH contents was performed with solid-phase extraction (SPE) using diatomaceous earth (Extrelut-20) columns coupled with propylsulfonic acid (PRS) columns, according to the previously described method of Janoszka et al. [21], with some modifications. Briefly, 5 g of grilled meat samples or bioaccessible fractions were homogenized with 10 g Na_2_SO_4_ in 15 mL 1 M cold NaOH solution for 3–6 h. Each of the samples was mixed with Extrelut refill material (diatomaceous earth, 17 g) and the mixture was loaded into a 20 mL Extrelut column, which was connected to a PRS SPE column. Dichloromethane (60 mL) was used to elute the PAH fraction. For clean-up, the dichloromethane extract was evaporated to dryness and re-dissolved in 1 mL of n-hexane. The resulting solution was then placed on top of a column containing 10 g of silica gel (preconditioned with 25 mL n-hexane). The column was eluted with 60 mL n-hexane: dichloromethane (60:40 *v*/*v*), and the eluate was collected. The purified PAH’s fraction was concentrated to dryness. Acetonitrile (250 µL) was used to dissolve the dried PAH residue for HPLC analysis.

### 2.6. Preparation of Standard Solutions of PAHs

The stock standard solutions of 100 µg/mL were prepared by diluting the purchased standards in HPLC grade acetonitrile. The working solutions at different concentrations (0.1–100 ng/mL) were then prepared by diluting the stock solutions with the same solvent. The stock and working standard solutions were stored at −20 °C prior to use. PAH standard solutions at eight concentrations of 0.1, 0.5, 1, 10, 25, 50, 75 and 100 ng/mL were injected into the HPLC to construct the standard calibration curve used for PAH quantification.

### 2.7. Determination of PAHs in the Sample Using HPLC

Quantitative analysis of 15 PAHs was performed using a previously described method [18], with modifications. A Waters (Milford, MA, USA) high performance liquid chromatography (HPLC) equipped with ultraviolet (UV, 254 nm) and fluorescence (FLD, λ_ex_ = 360 nm, λ_em_ = 460 nm) detectors was used. Compounds were separated on a PAH column (5 µm, 250 mm × 4.6 mm; Hichrom, Reading, UK), with water (solvent A) and acetonitrile (solvent B) flowing at 1.5 mL/min as a mobile phase. The solvent system started with 50% B which was maintained for 5 min and linearly increased to 100% B in 25 min. In the next 10 min, the solvent system resumed at 50% B. The excitation and emission wavelengths were 276/330 nm for Na, Ac and F, 296/400 nm for Phe, BaA, Ch, BkF and BaP, 340/425 nm for A, Fl, P, BbF, DhA and BgP, and 246/488 nm for IP. Before injection into the HPLC system, each sample of PAH solution was passed through 0.45 µm syringe filter. The PAHs were identified by comparing their retention times with those of the standard PAHs. The concentrations of PAHs in grilled meat samples and bioaccessible fractions were determined using an external calibration curve of PAH standards.

### 2.8. Method Validation

The HPLC/UV-FLD method for quantification of PAHs was validated with respect to parameters including linearity, limit of quantification (LOQ), limit of detection (LOD) and recovery, as previously described by Lee et al. [2]. The linearity was determined by regression analysis, where calibration curves for individual PAHs were constructed by plotting average peak area against the concentration, and a regression equation was generated. Meanwhile, LOD and LOQ were defined as the lowest concentration of the sample determined by the analytical method to obtain the signal-to-noise ratios of 3:1 and 10:1, respectively. Eight replicates of PAH standard mixtures were injected into the HPLC system to determine the LOD and LOQ. To evaluate the accuracy and efficiency of the extraction method, a recovery test was carried out. The experiment was performed in seven replicates by spiking 40 ng of PAHs mixed standards in each grilled sample (5 g). The spiked samples were stirred for one hour at room temperature before extraction, and the un-spiked ones were used as a control. Recoveries were calculated from the differences of concentrations between the spiked and un-spiked samples in the amount of PAHs. According to European Union criteria for food contaminant control, a recovery range of 50–170% is considered acceptable [22].

### 2.9. Determination of Fat Content in Grilled Meat Samples

All the meat samples were analyzed for fat content using Soxhlet method (AOAC Method 960.39; [23]), with some modifications. Briefly, 2 g of the meat sample were added to an extraction thimble, then dried in an oven at 125 °C for about 80 min. A pre-dried boiling flask was weighed, and the fat content of the samples was extracted using petroleum ether. After 6 h of extraction, the fat content was calculated using the following equation:

(2) $$\%\;Fat\;\left(dry\;weight\;basis\right)=\frac{g\;of\;fat\;extracted}{g\;of\;dried\;sample}\times 100\%\;$$

### 2.10. Dietary Intake of PAHs through Grilled Beef and Chicken Consumption

Based on the bioaccessibility of PAHs from this study, dietary intake (*DI*) of PAHs through grilled beef and chicken consumption was calculated using the following equation:

(3) $$DI=\frac{C\times M\times IR}{BW}\;$$

where *C* is the average concentration of total PAHs in µg/kg, *M* is food consumption rate, *IR* is the bioaccessibility of PAHs determined in this study and *BW* is the average body weight of the general population [24].

### 2.11. Statistical Analysis

Data were expressed as mean ± SD for seven replicates. One-way analysis of variances (ANOVA) with Tukey’s post hoc test was performed to determine the significant differences in bioaccessibility of PAHs at different GIT segments, as well as among the grilled meat samples with different degrees of doneness and fat contents, at a confidence level of 95%. Pearson correlation was used to analyze the relationship between bioaccessibility of PAHs and degrees of doneness and fat content of grilled meat samples. Statistical analysis was performed using MS Excel (version 2013, Microsoft, Redmond, WA, USA) and Minitab (Version 16, Minitab Inc, State College, PA, USA) software.

## 3. Results and Discussion

### 3.1. Evaluation of Analysis Procedure Performance

**Figure 1:**
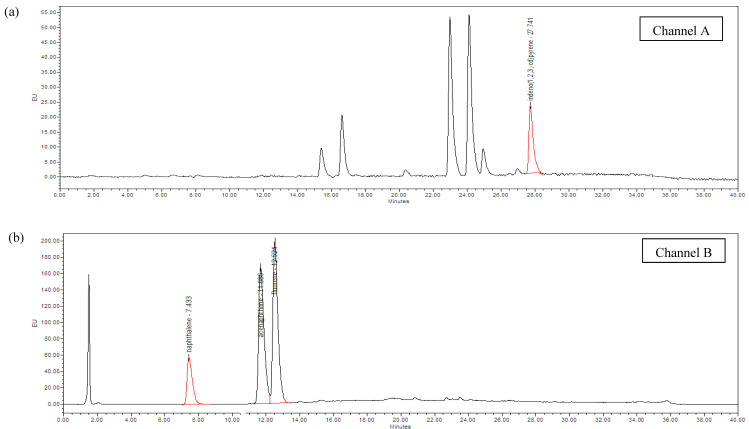

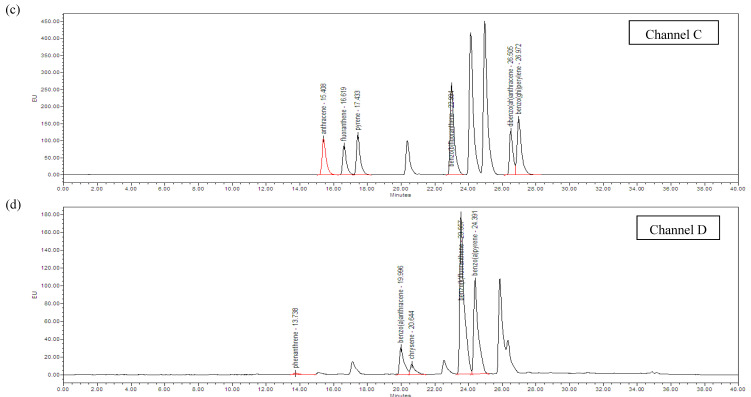
HPLC/UV-FLD chromatograms of PAHs’ standard mixture. The PAHs were detected at four different channels of the fluorescence detector; (**a**) Channel A: λ_ex_ = 246 nm, λ_em_ = 488 nm; (**b**) Channel B: λ_ex_ = 276 nm, λ_em_ = 330 nm; (**c**) Channel C: λ_ex_ = 296 nm, λ_em_ = 400 nm; (**d**) Channel D: λ_ex_ = 340 nm, λ_em_ = 425 nm. Red line showed the detected PAHs.

Optimization of HPLC procedures for determining the 15 analyzed PAHs was done using an HPLC system equipped with UV and FL detectors. Emission and excitation wavelengths were selected based on previous studies [25,26] to obtain high sensitivity for the detection of the analyzed compounds. Identification of the PAHs in grilled meat samples was done based on the comparison of their retention times with those of PAH standards. Figure 1 shows the chromatogram of the 15 PAHs’ standards, which were detected at four excitation and emission wavelengths of fluorescence detector. As observed on the chromatograms, the elution order of the PAHs was in the order of increasing molecular weight. Compounds with the same molecular weight were also separated well. Furthermore, no interfering peak appeared in the areas of concern, and therefore the chromatogram was acceptable for the quantification of the analyzed PAHs.

Analytical performance of the developed HPLC/UV-FLD method was assessed through the estimation of LOD, LOQ, linearity and recovery. The results of LOD, LOQ and linearity were displayed in Table 2. The LODs and LOQs for the 15 analyzed PAHs ranged from 0.025–5 ng/g and 0.075–15 ng/g, respectively. This suggests that the developed method offered high sensitivity for the detection of the PAH compounds in the samples [23]. The LOD and LOQ, reported by Purcaro et al. [27] for the method analysing BaP, BaA, BbF and Ch, were ≤0.3 and ≤0.9 μg/kg, respectively. On the other hand, the regression coefficient of the standard curves between the peak area and concentrations of 15 PAHs ranged from 0.970 to 0.987, showing acceptable linearity of the standard curves.

The recoveries of the PAHs spiked in the grilled meat samples are shown in Table 3. The results revealed a wide range of PAH recoveries in the grilled meat samples, with 13.68–132.64% and 48.79–98.85% for grilled beef and chicken samples, respectively. As compared to grilled beef, grilled chicken samples show a relatively lower recovery of PAHs. This may be due to differences in the type of meat and hence the chemical compositions, which eventually affect the efficiency of PAH extraction. In addition, the present findings vary from previous reports analyzing the same PAHs in thermally prepared meats [26,28,29]. This may be attributed to the different sample matrix as well as the different PAH extraction and purification procedures [16]. In addition, the complex food matrices, complex nature of the marinades used and also absence of a large amount of other materials apart from PAHs may also disrupt the analytical determination and may decrease the extraction efficiency of PAHs [30]. According to the European Union’s criteria for food contaminant control (SANCO document No. 12571/2013) [22], which is usually referred to for pesticide residue and other food contaminants, a range of 70–120% should be achieved recovery of analytes. Therefore, for subsequent analysis, only the PAHs with recovery in the range of 70–120% were considered to ensure reliable PAH quantification in the present study, including acenaphthene, phenanthrene, anthracene, pyrene, benzo(k)fluoranthene, benzo(a)pyrene and indeno(1,2,3-cd)pyrene.

### 3.2. Bioaccessibility of PAHs in Grilled Meat in Different Parts of Digestive System

**Figure 2:**
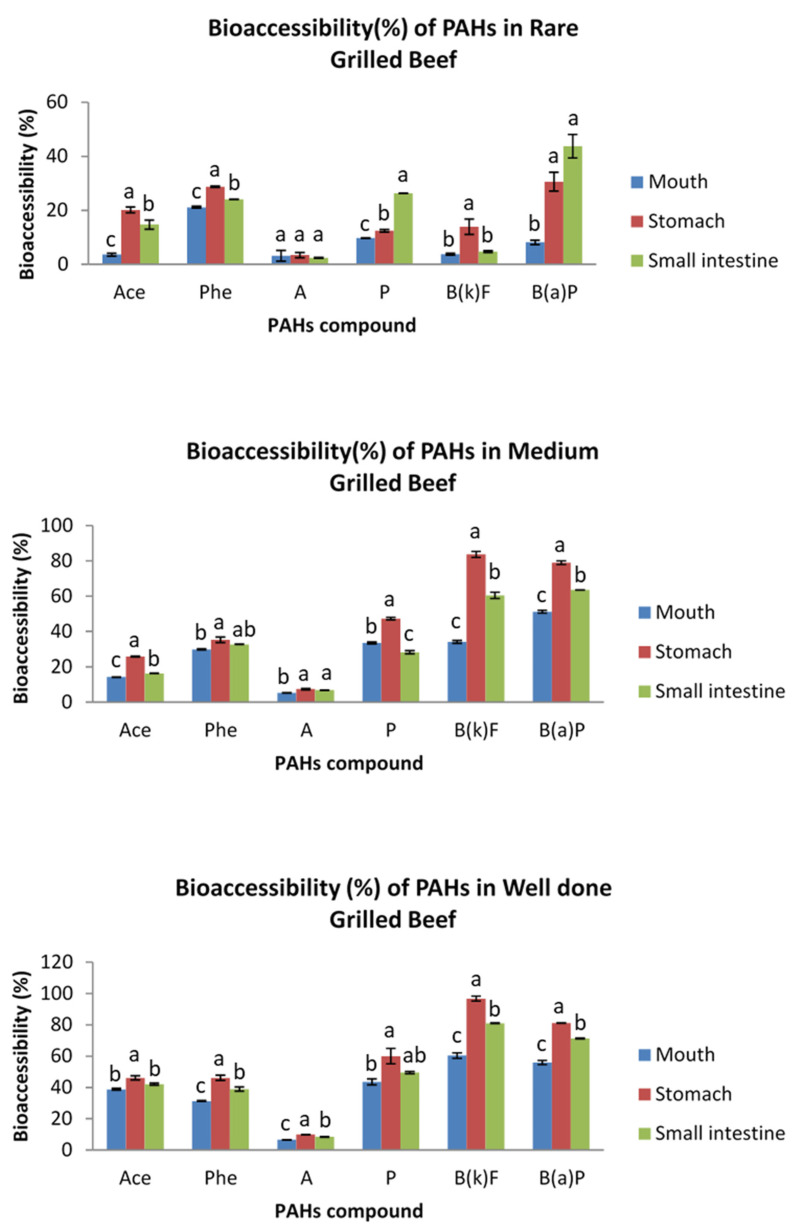
Bioaccessibility (%) of PAHs in rare, medium and well-done grilled beef samples in different parts of digestive system. Different letter indicates significant difference in PAH’s bioavailability among the different parts of the digestive system. Ace, acenaphthene; Phe, phenanthrene; A, anthracene; P, pyrene; B(k)F, benzo(k)fluoranthene; B(a)P, benzo(a)pyrene.

**Figure 3:**
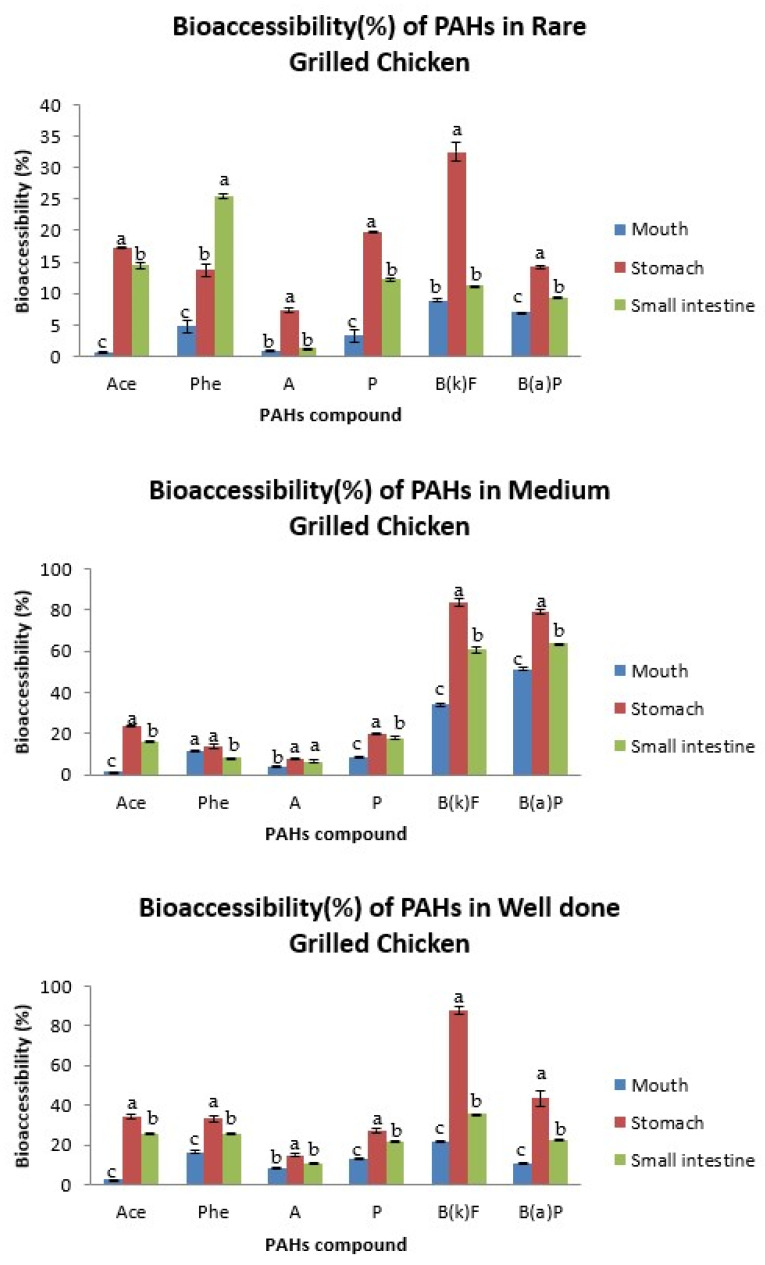
Bioaccessibility (%) of PAHs in rare, medium and well-done grilled chicken samples in different parts of digestive system. Different letter indicates significant difference in PAH’s bioavailability among the different parts of the digestive system. Ace, acenaphthene; Phe, phenanthrene; A, anthracene; P, pyrene; B(k)F, benzo(k)fluoranthene; B(a)P, benzo(a)pyrene.

The bioaccessibility of PAHs in grilled meats was assessed at the different segments of the GIT, namely mouth, stomach and small intestine for the salivary, gastric and intestinal digestion, respectively. The bioaccessibility results for grilled beef are presented in Figure 2, while Figure 3 displays the results for grilled chicken samples. The bioaccessibility of indeno(1,2,3-cd)pyrene was not able to be determined, as its concentration in the bioaccessible fractions of all studied samples was below the limit of detection. This could be attributed to its low polarity that hindered its effective extraction by common solid phase extraction materials [31]. Besides, as reported by Yu et al. [17], the high-molecular weight (HMW) of PAHs such as indeno(1,2,3-cd)pyrene, dibenzo(ah)anthracene and benzo(ghi)perylene is usually undetected or below the limits of detection, particularly in livestock and poultry samples.

A wide range of PAHs’ bioaccessibility was detected in the mouth, stomach and small intestine. The bioaccessibility of the analyzed PAHs in grilled beef ranged from 3.18 to 60.44%, 3.40 to 96.71% and 2.38 to 81.02% in the salivary, gastric and intestinal conditions, respectively. Meanwhile, those in grilled chicken ranged from 0.67 to 51.13%, 7.36 to 87.77% and 1.26 to 63.55%, respectively. Overall, the results showed that there was variation between the PAH’s bioaccessibility in grilled beef and chicken, with those in grilled beef exhibiting greater bioaccessibility. This could be attributed to the variation in PAH’s levels in grilled beef and grilled chicken, in agreement with the previous study [32], in which the concentration of PAHs was higher in charcoal grilled beef as compared to charcoal grilled chicken. The different concentration of PAHs in grilled beef and grilled chicken could be contributed to by the difference in fat content, myoglobin content and marbling between the beef and chicken meat muscles [33].

Regardless of the meat type and cooking doneness, the PAHs in grilled beef and chicken showed the highest level of bioaccessibility in the stomach, followed by the small intestine. These PAHs were only slightly bioaccessible in the mouth. The low bioaccessibility in the mouth was due to the short residence time that the PAHs spent in the mouth (5 min), thus, salivary digestion may not contribute significantly to PAH’s release from the grilled meat. Meanwhile, the high bioaccessibility of PAHs in the stomach was due to the low pH (1.3) of gastric juice, which helped to digest the grilled meat and, hence, release more PAHs. However, the subsequent neutralizing effect by bile salts in the small intestine resulted in decreased bioaccessibility of PAHs in the intestinal digestion step. These results are contrary to the previous findings [15,18], wherein the bioaccessibility of PAHs in the intestinal condition was significantly higher than the gastric condition. The difference in the findings could be attributed to the different incubation time during the in vitro digestion of the food. The incubation time during the gastric and intestinal digestion used in the current study was respectively higher and lower as compared to the previous studies [15,18]. Besides, factors such as enzyme concentration, pH, type of enzymes used, volume of digestive juices, amount and water content of samples in the digestive model can also contribute to the variation in results [20].

### 3.3. Bioaccessibility of PAHs in Grilled Meat at Different Degree of Doneness

**Figure 4:**
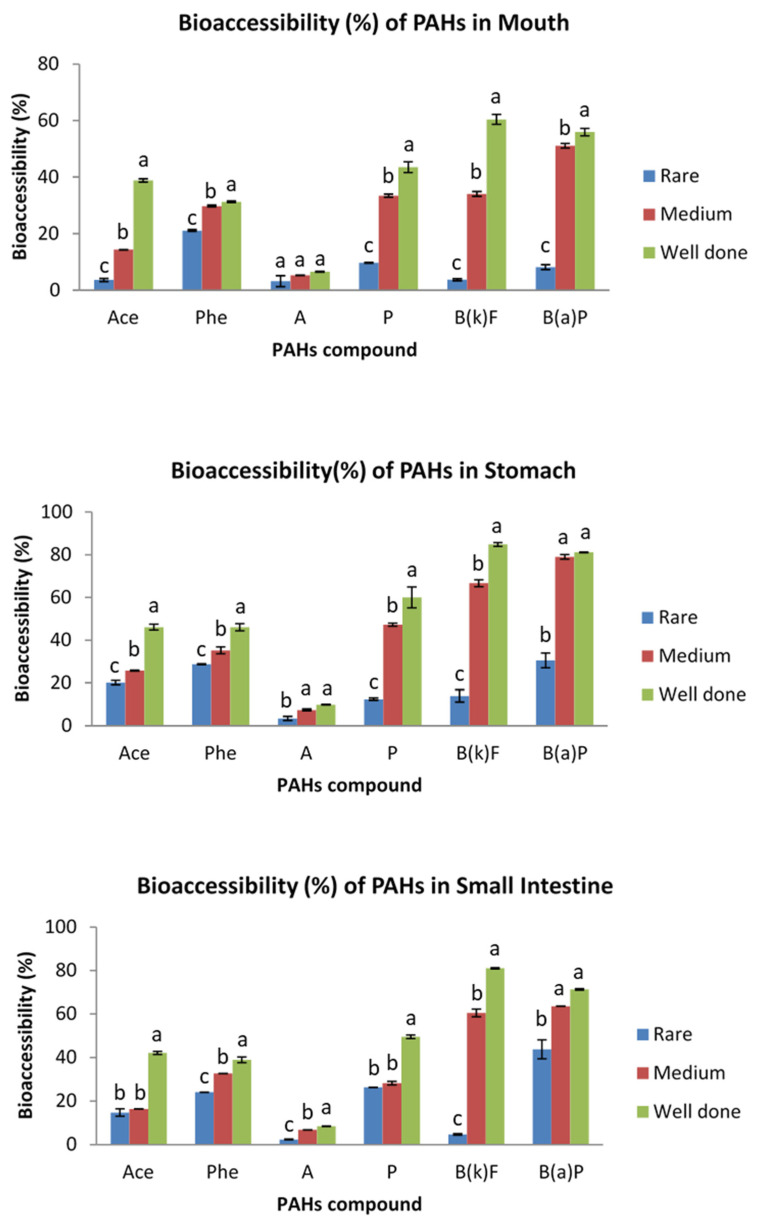
Bioaccessibility (%) of PAHs in grilled beef at different degrees of doneness in mouth, stomach and small intestine. Different letter indicates significant differences in bioavailability of PAHs in grilled beef of different doneness. Ace, acenaphthene; Phe, phenanthrene; A, anthracene; P, pyrene; B(k)F, benzo(k)fluoranthene; B(a)P, benzo(a)pyrene.

**Figure 5:**
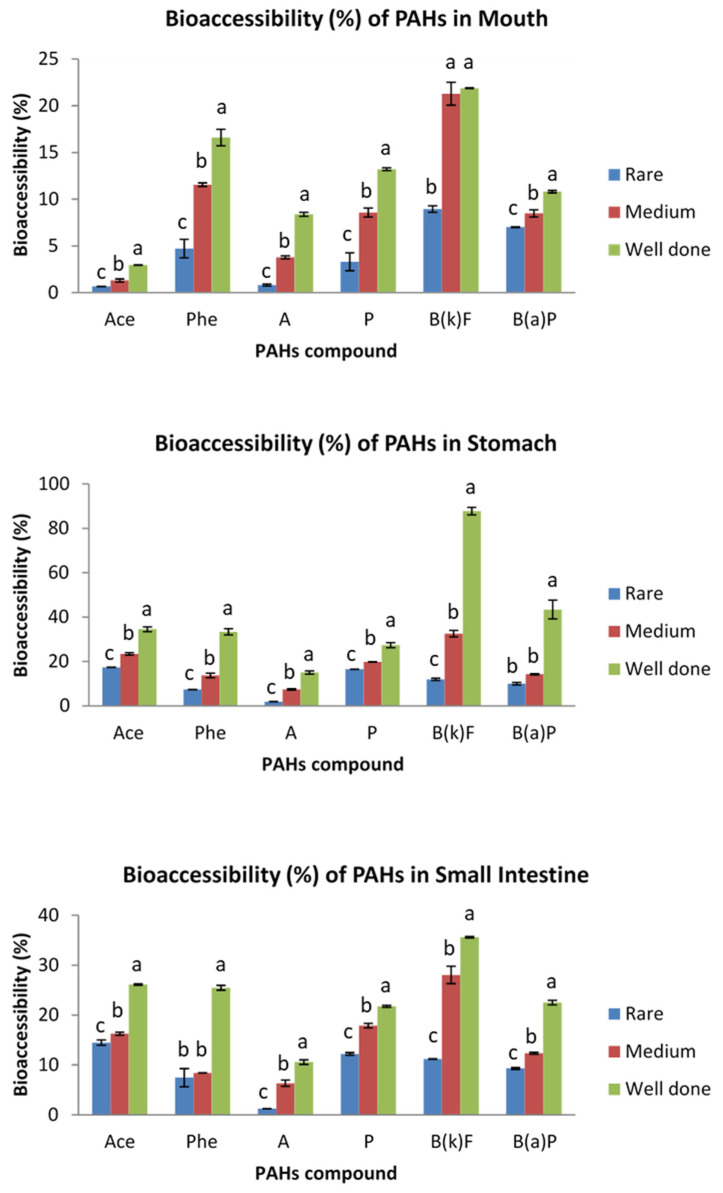
Bioaccessibility (%) of PAHs in grilled chicken at different degrees of doneness in mouth, stomach and small intestine. Different letter indicates significant differences in bioavailability of PAHs in grilled chicken of different doneness. Ace, acenaphthene; Phe, phenanthrene; A, anthracene; P, pyrene; B(k)F, benzo(k)fluoranthene; B(a)P, benzo(a)pyrene.

Degree of doneness is one of the main issues for PAH’s formation in cooked meat. In order to explain the effect of degree of doneness on the bioaccessibility of PAHs, the chicken and beef samples were grilled using charcoal at three levels of doneness, namely rare, medium and well-done. The degree of doneness was based on the browning of the sample meats’ surfaces and total cooking time [19]. The bioaccessibility of PAHs in grilled beef and chicken at varying degrees of doneness is presented in Figure 4 and Figure 5, respectively.

The bioaccessibility of the analyzed PAHs in rare, medium and well-done grilled beef was in the range of 2.38–43.75%, 5.27–63.55% and 6.51–84.83%, respectively. Meanwhile, the ranges for rare, medium and well-done grilled chicken were 0.67–17.33%, 1.30–32.50% and 2.95–87.77%, respectively. Overall, the results demonstrated that increasing the degree of meat doneness increased the average bioaccessibility of PAHs from the meat matrix. Bioaccessibility of PAHs was higher in well-done grilled meat as compared to rare and medium grilled samples. These results can be explained by the fact that well-done meats were exposed to heat longer than rare and medium cooked samples, resulting in a charred surface with a high PAH level and hence high bioaccessibility, in line with previous studies reporting an increase in carcinogenic PAHs with increasing duration of cooking, or “doneness”, of the meat [19,33].

### 3.4. Effect of Fat Content on the Bioaccessibility of PAHs in Grilled Beef and Chicken

**Figure 6:**
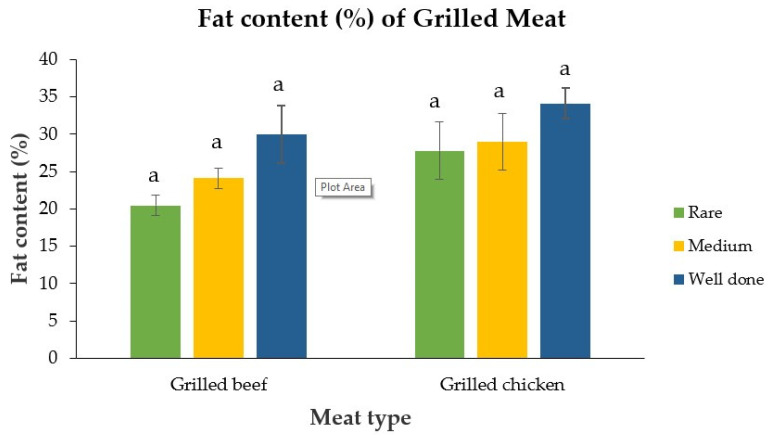
Fat content (%) of rare, medium and well-done grilled beef and chicken samples. Similar letter indicates no significant differences.

The fat content of the meat grilled to different levels of doneness was assessed and a linear regression analysis was performed to evaluate the relationship between the fat content and the bioaccessibility of PAHs in grilled meat. The percentage (%) of fat in the grilled beef and chicken at different degrees of doneness is presented in Figure 6. The results show that the fat contents of the meat samples increased with the degree of doneness. This could be explained by the concentrating effect of fat as moisture is lost with the increasing level of doneness.

**Figure 7:**
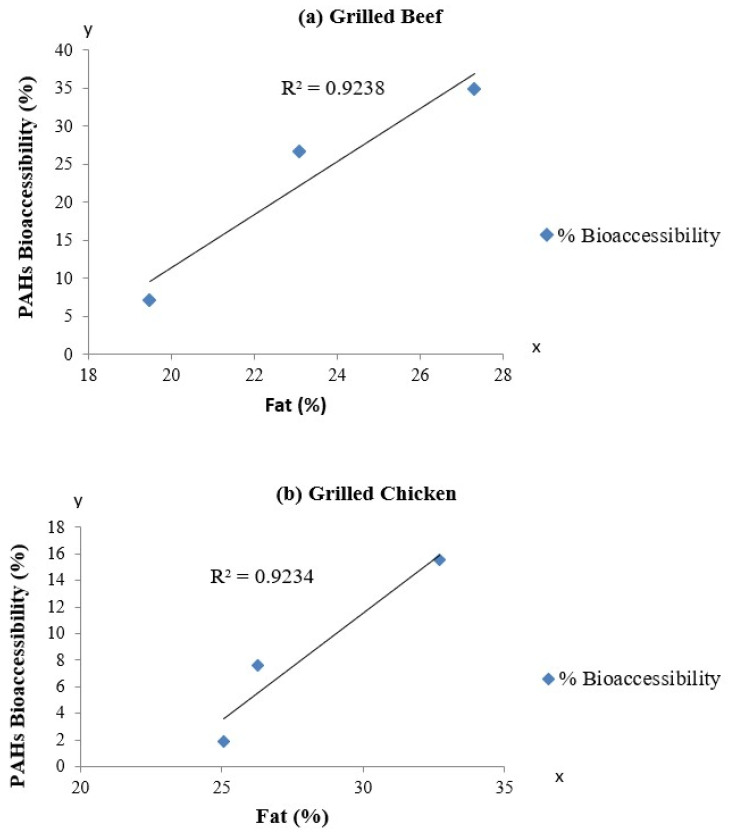
Relationship between the bioaccessibility of PAHs and the fat content in (**a**) grilled beef and (**b**) grilled chicken samples.

Furthermore, the correlation between the fat contents and bioaccessibility of PAHs in grilled beef and chicken is shown in Figure 7, and the results revealed positive correlations among these two parameters. These results are in accordance with findings, reported by Yu et al. [17], in which positive linear relationships between the bioaccessibility of PAHs and the lipid contents in animal-based foods were observed. Since PAHs are highly lipophilic molecules, they were expected to accumulate in the fat contained in food material. During digestion, fat containing PAHs will be emulsified by bile and brought into the liquid phase, becoming accessible for uptake. Therefore, it can be suggested that the constituents of foods, particularly the fat content, exhibit a great influence on the bioaccessibility of PAHs. The bioaccessibility of PAHs can hence be estimated via the calculation of the fat contents in food.

### 3.5. Estimation of Daily Dietary Intake (DI) of PAHs via Consumption of Grilled Beef and Chicken

Other than occupational exposure, diet makes a significant contribution (>70% in nonsmokers) to PAH intake [14]. Intake via grilled, barbecued and smoked meat contributes a substantial proportion of total dietary intake of PAHs, since these foods are gaining popularity nowadays, both in restaurants and at home [6]. In this present study, daily dietary intake (*DI*) of PAHs via consumption of grilled beef and chicken was estimated through a formula adopted from Nasher et al. [20]. The daily DI of PAHs via consumption of grilled beef and chicken is presented in Table 4 and Table 5, respectively. For grilled beef, the PAH with the highest daily *DI* was benzo(k)fluoranthene (1.31 µg/day), via the consumption of medium-grilled meat and intestinal digestion. For grilled chicken, benzo(k)fluoranthene also exhibited the highest daily *DI* (23.22 µg/day), however, unlike grilled beef, it was through the consumption of well-done grilled chicken and gastric digestion. In contrast to the report of dos Santos Fogaça et al. [16], wherein the cooking process did not influence the PAH’s *DI*, the results of the current study revealed that the *DI* of the PAHs increased with the increase in cooking time (degree of meat doneness). Variation in the results could be attributed to the different food matrices which contributed to the different PAHs’ bioaccessibility, since bioaccessibility was integrated into the *DI* estimation. Overall, the results in the present study revealed consumption of medium grilled beef and well-done grilled chicken played a very important role in PAH ingestion, especially the intake of benzo(k)fluoranthene.

## 4. Conclusions

In this study, the bioaccessibility of PAHs in grilled beef and chicken with different degrees of doneness in the different segments of the GIT was investigated. The relationship between the fat contents of grilled meats and the bioaccessibility of PAHs was also studied. For the PAHs detected, the results showed variation between the PAH’s bioaccessibility in grilled beef and chicken, with those in grilled beef exhibiting relatively higher bioaccessibility. The highest bioaccessibility of PAHs resulted from gastric digestion, followed by intestinal and salivary digestions. In relation to the degree of doneness, the bioaccessibility of PAHs increased with the increase in meat doneness, and they were positively correlated to the fat contents of the grilled meats. The estimated daily intake of PAHs was also found to be affected by the degree of meat doneness, which could pose cancer risk via long term ingestion of grilled meats. Overall, the findings of this study reveal the influence of meat doneness (cooking time) on the bioaccessibility and bioaccumulation of PAHs. These findings can be employed in health risk assessment of human exposure to PAHs due to consumption of grilled meat at different degrees of doneness.

## Figures and Tables

**Table 1:** Amount of ingredients used for the preparation of marinades (for 1 kg meat).

Ingredient	Amount (g)
Cumin	50
Shallots	150
Coriander powder	100
Lemongrass	100
Turmeric powder	50
Sugar	100
Salt	10
Cooking oil	10

**Table 2:** Linear equations, coefficients of regression, limit of detection (LOD) and quantification (LOQ) obtained for quantification of PAHs.

Standards	Concentration Range (ng/g)	Linear Equation	Regression Coefficient (R2)	LOD (ng/g)	LOQ (ng/g)
Naphthalene	0.1–100	y = 6.4792 x + 4.7790	0.981405	1.5	4.5
Acenaphthene	0.1–100	y = 1.7493 x − 3.4145	0.987578	0.6	1.8
Fluorene	0.1–100	y = 1.8027 x − 2.8453	0.987042	0.7	2.1
Phenanthrene	0.1–100	y = 8.882 x − 6.6481	0.965233	5	15
Anthracene	0.1–100	y = 6.2336 x − 2.5416	0.984748	0.7	2.1
Fluoranthene	0.1–100	y = 5.3291 x − 2.1768	0.981000	0.8	2.4
Pyrene	0.1–100	y = 7.4040 x − 3.8008	0.979026	0.6	1.8
Benzo(a)anthracene	0.1–100	y = 1.4339 x − 3.8190	0.977714	0.2	0.6
Chrysene	0.1–100	y = 4.6118 x − 1.4542	0.970663	1	3
Benzo(b)fluoranthene	0.1–100	y = 1.4982 x − 1.6602	0.987029	0.8	2.4
Benzo(k)fluoranthene	0.1–100	y = 6.2506 x − 2.1473	0.983345	0.025	0.075
Benzo(a)pyrene	0.1–100	y = 4.2828 x − 7.3741	0.986418	0.5	1.5
Dibenzo(ah)anthracene	0.1–100	y = 4.4584 x − 6.9380	0.986124	0.04	0.12
Benzo(ghi)perylene	0.1–100	y = 1.4985 x − 1.2393	0.977307	0.03	0.09
Indeno(1,2,3-cd) pyrene	0.1–100	y = 2.0460 x − 1.3854	0.983383	3	9

**Table 3:** Recoveries of PAHs spiked in grilled beef and chicken meat samples.

Standards	Replication	Spiked Concentration (ng/g)	Recovery of PAHs (%)	
			Grilled Beef	Grilled Chicken
Naphthalene	7	8	94.82 ± 6.37	60.85 ± 0.11
Acenaphthene	7	8	94.83 ± 1.46	94.83 ± 6.24
Fluorene	7	8	13.68 ± 0.16	98.85 ± 1.79
Phenanthrene	7	8	89.54 ± 8.65	80.69 ± 5.56
Anthracene	7	8	97.98 ± 4.10	74.98 ± 0.39
Fluoranthene	7	8	57.88 ± 3.13	81.1 ± 3.13
Pyrene	7	8	80.35 ± 9.56	85.21 ± 1.47
Benzo(a)anthracene	7	8	74.96 ± 2.67	48.79 ± 1.80
Chrysene	7	8	43.37 ± 0.93	64.13 ± 1.20
Benzo(b)fluoranthene	7	8	68.47 ± 2.81	56.89 ± 0.35
Benzo(k)fluoranthene	7	8	94.61 ± 2.87	75.07 ± 3.53
Benzo(a)pyrene	7	8	91.31 ± 8.15	91.31 ± 8.15
Dibenzo(ah)anthracene	7	8	132.64 ± 3.14	64.34 ± 2.90
Benzo(ghi)perylene	7	8	87.84 ± 1.69	68.88 ± 9.79
Indeno(1,2,3-cd) pyrene	7	8	79.42 ± 1.39	81.35 ± 1.27

**Table 4:** Daily dietary intake (*DI*) of PAHs via consumption of grilled beef.

PAH	Dietary Daily Intake (*DI*, µg/day)								
	Mouth			Stomach			Small Intestine		
	Rare	Medium	Well-Done	Rare	Medium	Well-Done	Rare	Medium	Well-Done
Acenaphthene	0.04	0.27	0.19	0.19	0.34	0.22	0.52	0.62	0.57
Phenanthrene	0.28	0.39	0.31	0.40	0.47	0.44	0.42	0.62	0.52
Anthracene	0.04	0.04	0.03	0.07	0.10	0.09	0.08	0.13	0.11
Pyrene	0.13	0.16	0.35	0.45	0.64	0.38	0.59	0.81	0.67
Benzo(k)fluoranthene	0.05	0.18	0.06	0.46	1.13	0.82	0.82	1.31	1.09
Benzo(a)pyrene	0.11	0.41	0.59	0.69	1.07	0.86	0.75	1.10	0.9

**Table 5:** Daily dietary intake of PAHs via consumption of grilled chicken.

PAH	Dietary Daily Intake (*DI*, µg/day)								
	Mouth			Stomach			Small intestine		
	Rare	Medium	Well-Done	Rare	Medium	Well-Done	Rare	Medium	Well-Done
Acenaphthene	0.17	0.34	0.78	4.56	6.18	9,13	3.83	4.30	6.90
Phenanthrene	1.25	0.30	4.38	1.96	3.63	8.81	1.97	2.21	6.73
Anthracene	0.21	1.02	2.21	0.50	1.94	3.97	0.33	1.67	2.79
Pyrene	0.87	2.26	3.49	4.36	5.24	7.24	3.23	4.73	5.75
Benzo(k)fluoranthene	2.36	5.62	5.78	3.14	8.59	23.22	2.96	7.41	9.42
Benzo(a)pyrene	1.85	2.24	2.85	2.64	3.76	11.48	2.46	3.27	5.95

## Data Availability

Data is contained within the article or supplementary material.

## Supplementary Materials

The following are available online at https://www.mdpi.com/article/10.3390/ijerph19020736/s1, Table S1: Cooking parameters for meat at three different degrees of doneness, Table S2: Composition of digestive juices used per 100 mL of ultrapure water, Figure S1: Schematic diagram of the in vitro digestive procedure (Adapted from [17]).
