# Supplementary material for: Bioaccessibility of Polycyclic Aromatic Hydrocarbons (PAHs) in Grilled Meat: The Effects of Meat Doneness and Fat Content
Source: Int J Environ Res Public Health. 2022 Jan 10;19(2):736. doi: 10.3390/ijerph19020736 (PMC8775937; doi:10.3390/ijerph19020736)
Supplement: Supplementary file 1 [file ijerph-19-00736-s001.zip › ijerph-1419886-supplementary.pdf]

Supplementary Materials

Table S1. Cooking parameters for meat at three different degrees of doneness.

**Samples**	**Degree of doneness**	**Cooking time (min)**	**Surface (browning/charring)**
Chicken	Rare	4	Slight
	Medium	5	Moderate
	Well done	6	Well browned/Blackened
Beef	Rare	6	Slight
	Medium	7	Moderate
	Well done	8	Well browned/Blackened

Table S2. Composition of digestive juices used per 100 ml of ultrapure water.

Components	Digestive juices			
	Saliva (pH 6.8 ± 0.2)	Gastric (pH 1.30 ± 0.02)	Duodenal (pH 8.1 ± 0.2)	Bile (pH 8.2 ± 0.2)
Inorganic	- 90 mg KCl<br>- 20 mg KSCN<br>- 89 mg NaH_2PO_4<br>- 58 mg Na_2SO_4<br>- 30 mg NaCl<br>- 170 mg $NaHCO_3$	- 275 mg NaCl<br>- 27 mg NaH_2PO_4<br>- 82 mg KCl<br>- 31 mg NH_4Cl<br>- HCl 1 M	- 700 mg NaCl<br>- 339 mg $NaHCO_3$<br>- 8 mg KH_2PO_4<br>- 56 mg KCl<br>- HCl 1 M	- 526 mg NaCl<br>- 579 mg $NaHCO_3$<br>- 38 mg KCl<br>- HCl 1M
Organic	- 20 mg urea	- 65 mg glucose<br>- 2 mg glucuronic acid<br>- 8.5 mg urea<br>- 33 mg glucosamine hydrochloride<br>-100 mg BSA	- 100 mg urea<br>- 100 mg BSA	- 25 mg urea<br>- 180 mg BSA
Bioactive	- 29 mg α-amylase<br>- 1.5 mg uric acid<br>- 2.5 mg mucin	- 250 mg pepsin	- 900 mg pancreatin<br>- 150 mg lipase	- 3g bile salt

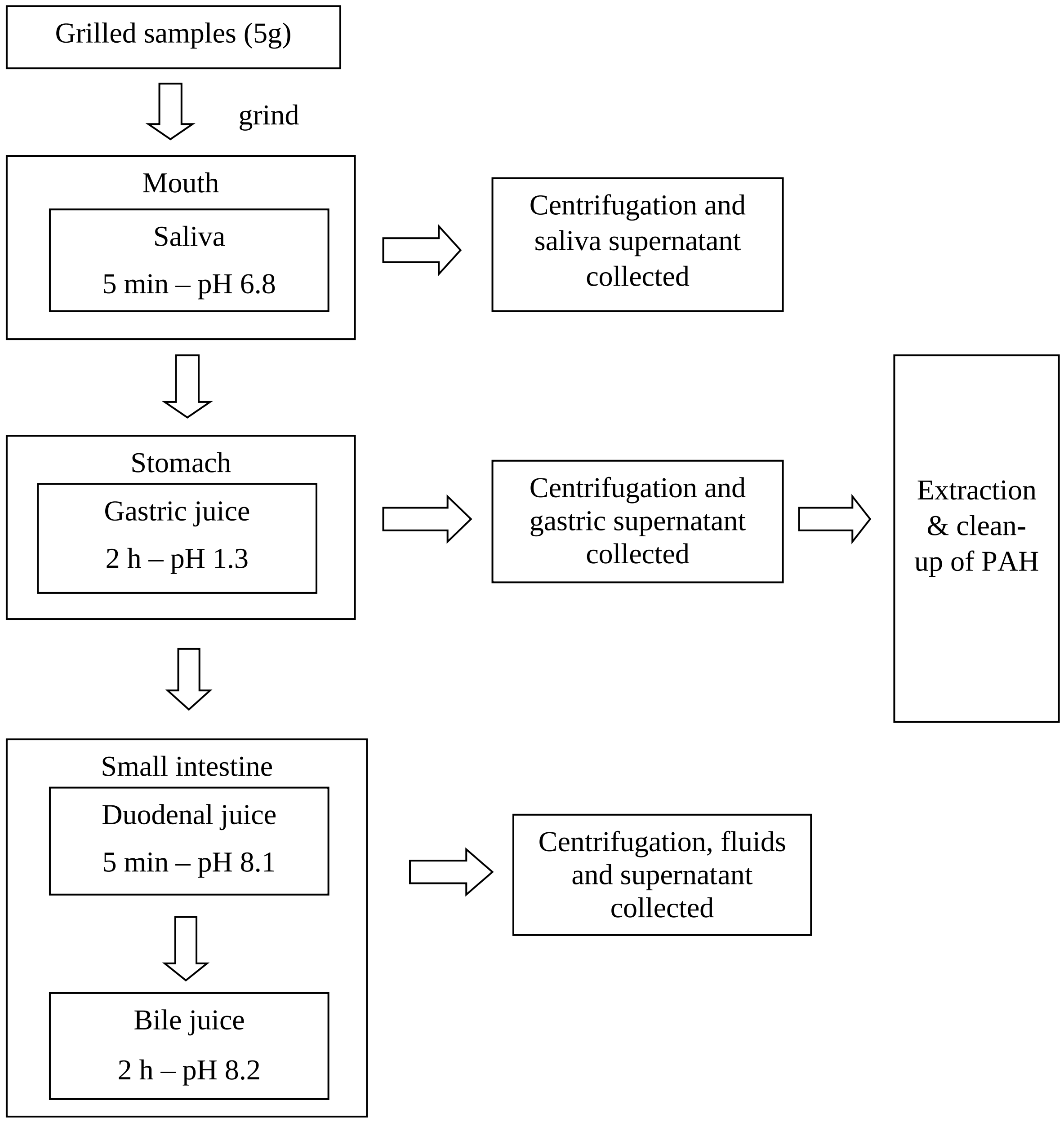

Figure S1. Schematic diagram of the *in vitro* digestive procedure (Adapted from [17]).
